# Endothelial Function and Arterial Stiffness Should Be Measured to Comprehensively Assess Obstructive Sleep Apnea in Clinical Practice

**DOI:** 10.3389/fcvm.2021.716916

**Published:** 2021-10-05

**Authors:** Jinmei Luo, Xiaona Wang, Zijian Guo, Yi Xiao, Wenhao Cao, Li Zhang, Linfan Su, Junwei Guo, Rong Huang

**Affiliations:** ^1^Department of Pulmonary and Critical Care Medicine, Peking Union Medical College Hospital, Chinese Academy of Medical Sciences, Peking Union Medical College, Beijing, China; ^2^Department of Clinical Laboratory, Peking Union Medical College Hospital, Chinese Academy of Medical Sciences, Peking Union Medical College, Beijing, China

**Keywords:** obstructive sleep apnea, vascular endothelial function, arterial stiffness, reactive hyperemia index, augmentation index

## Abstract

**Objective:** An effective clinical tool to assess endothelial function and arterial stiffness in patients with obstructive sleep apnea (OSA) is lacking. This study evaluated the clinical significance of subclinical markers for OSA management in males without serious complications.

**Patients/Methods:** Males without serious complications were consecutively recruited. Clinical data, biomarker tests, reactive hyperemia index (RHI), and augmentation index at 75 beats/min (AIx75) measured by peripheral arterial tonometry were collected. An apnea hypopnea index (AHI) cutoff of ≥15 events/h divided the patients into two groups.

**Results:** Of the 75 subjects, 42 had an AHI ≥15 events/h. Patients with an AHI ≥15 events/h had higher high-sensitivity C-reactive protein, tumor necrosis factor-alpha (TNF-α), vascular endothelial growth factor, and AIx75 values than the control group but no statistical difference in RHI was observed. After controlling for confounders, TNF-α was negatively correlated with the average oxygen saturation (*r* = −0.258, *P* = 0.043). RHI was correlated with the rapid eye movement (REM) stage percentage (*r* = 0.306, *P* = 0.016) but not with AHI (*P* > 0.05). AIx75 was positively correlated with the arousal index (*r* = 0.289, *P* = 0.023) but not with AHI (*r* = 0.248, *P* = 0.052).

**Conclusions:** In males with OSA without severe complications, TNF-α and AIx75 are independently related to OSA. The role of RHI in OSA management requires further elucidation. These markers combined can comprehensively evaluate OSA patients to provide more evidence for the primary prevention of coronary heart disease and treatment response assessment.

## Introduction

Obstructive sleep apnea (OSA) is the most common sleep breathing disorder. A literature-based analysis in 2019 indicated that ~936 million patients had OSA in China, which was the country with the largest affected population (1). Recurrent apnea and hypopnea induced by OSA lead to intermittent hypoxia and fragmented sleep and affect people's quality of life and health. OSA is associated with many diseases including hypertension, cardiovascular disease, arrhythmia, heart failure, stroke, etc. (2–5). Even mild OSA without comorbidities may lead to vascular endothelial dysfunction and elevated arterial stiffness (6–8), which characterizes the early stage of arterial atherosclerosis and can predict cardiovascular events in the future (9–13). These subclinical abnormalities may provide additional evidence for the primary prevention of coronary heart disease and treatment response assessment.

An effective clinical tool to assess endothelial function and arterial stiffness in patients with obstructive sleep apnea (OSA) is lacking. Endothelial function can be measured by carotid intima media thickness (CIMT), flow-mediated dilation (FMD), and peripheral arterial tonometry (PAT) (14). Previous studies commonly used CIMT and FMD to measure vascular endothelial function (15, 16). However, increasing studies have applied the PAT technique to measure digital microvascular damage. The role of PAT in evaluating endothelial function in patients with OSA remains controversial (7, 17, 18). A lower reactive hyperemia index (RHI) derived from PAT indicates vascular endothelial dysfunction (9, 11). PAT is simple to perform in clinical practice and has a good reproducibility. A meta-analysis reported that FMD and PAT have similar prognostic values in predicting future cardiovascular events (19). Recent studies have shown that RHI was associated with the severity of OSA while others studies had negative results. Arterial stiffness also plays an important role in OSA evaluation and is considered a marker of atherosclerosis (20). A meta-analysis including 15 randomized controlled trials with 1,090 patients showed that continuous positive airway pressure (CPAP) can improve the augmentation index (AIx), which is a marker of arterial stiffness (21).

OSA is a heterogeneous disorder. It is unknown whether different subtypes of OSA have different vascular functions and stiffness. Gender can also influence endothelial function (22). Although some studies have applied PAT to explore the association between endothelial function and OSA, few studies have been performed in Chinese patients with OSA without severe comorbidities. Thus, we aimed to measure endothelial function and arterial stiffness using the PAT technique in Chinese males to assess the early vascular damage caused by OSA in patients without severe complications in clinical practice to provide more evidence for the primary prevention of coronary heart disease and treatment response assessment.

## Methods

### Participants

Participants with suspected sleep breathing disorders, hypertension, or healthy volunteers who agreed to undergo a polysomnography (PSG) in the sleep disordered breathing clinic at Peking Union Medicine College Hospital were consecutively and prospectively recruited from August 30th, 2019 to December 31st, 2019. Participants were excluded for any of the following reasons: younger than 25 years old or older than 60 years old; previously received treatment for sleep apnea with positive airway pressure therapy; self-reported chronic diseases including uncontrolled hypertension, liver diseases, kidney diseases, lung diseases, perivascular diseases, coronary atherosclerosis diseases, heart diseases, diabetes, thromboembolism diseases, tumor, nerve system diseases, and mental diseases; and acute infections in the past 2 weeks. All participants gave written informed consent, and this study was approved by local ethical review boards and was performed in accordance with the Declaration of Helsinki. Institutional review board approval was obtained from the ethics commission of Peking Union Medical College Hospital, Chinese Academy of Medical Sciences, China (protocol number JS-2013).

### Study Protocol

All the patients avoided vigorous exercises, smoking, alcohol, tea, coffee, and the night shift during the prior 24 h and were requested not to take naps or to take hypnotics or stimulants during the daytime before PSG. The patients' information including name, age, weight, height, and blood pressure before and after monitoring were recorded. Medical histories were taken by physicians at the sleep center. Comorbidities were self-reported. Hypertension was diagnosed when the patients had either a systolic blood pressure of ≥ 140 mmHg or a diastolic blood pressure of ≥ 90 mmHg at two different times or the current use of antihypertensive medications. Current smoking status was defined as patients who had been smoking in the past year.

### Polysomnography

Overnight PSG was performed using the standard PSG device Remlogic N7000 (USA, Medicare) in the sleep disordered breathing laboratory the same night. Electroencephalogram, electrooculograms, chin electromyogram, nasal detected by airflow pressure transducer, respiratory effort, electrocardiography, and position were recorded. All the sleep scoring and respiratory events were analyzed manually by a technician and checked by experienced sleep physicians. Manually sleep scoring and respiratory events were analyzed according to the AASM Manual 2.3 for the Scoring of Sleep and Associated Events in 2016 (23). Sleep scoring was performed for stages N1, N2, N3, rapid eye movement (REM), and wake. The apnea hypopnea index (AHI) was defined as the total number of apnea and hypopnea events each hour. The oxygen desaturation index (ODI) was defined as the frequency of instances when the blood oxygen levels decreased (desaturation) by 3% or more. The lowest pulse arterial oxygen saturation (LSpO_2_) was defined as the lowest pulse oxygen saturation during sleep. T90 (the percentage of SpO_2_ lower than 90%) was defined as the percentage of time that the oxygen saturation was lower than 90% during the monitoring time. The arousal index (AI) was defined as average number of arousals per hour of total sleep time. According to the American Academy of Sleep Medicine practice guidelines (24), we used an AHI of ≥15 events/h as a cutoff to divide the patients into two groups: the AHI <15 events/h group and the AHI ≥15 events/h group. OSA severity was defined as follows: mild OSA, 15 events/h > AHI ≥5 events/h; moderate OSA, 30 events/h > AHI ≥15 events/h; and severe OSA, AHI ≥30 events/h; the control group was defined as AHI <5 events/h.

### Digital Vascular Measurements

We used an automated device (EndoPAT2000; Itamar Medical, Caesarea, Israel) to measure the endothelial function and arterial stiffness the morning after the PSG. The participants were asked to lie on their backs for at least 30 min after blood was drawn, and the cuffs were placed on one arm 2 cm above the cubital fossa. PAT probes were placed on each index or middle finger. The baseline pulse amplitude was recorded for 5 min. Arterial flow was weakened until the pulse signal disappeared on one side for 5 min by inflating the cuffs to at least 200 mmHg (or 60 mmHg above the systolic pressure). After 5 min, the cuffs were deflated to induce reactive hyperemia, and the PAT signals were recorded for another 5 min. The contralateral fingers were used to control for systemic changes. The PAT ratio was the ratio of the post-deflate pulse amplitude 90–120 s after cuff release to the baseline mean pulse amplitude. This result was divided by the corresponding ratio from the control finger. Finally, the RHI was calculated automatically by computer software. A higher RHI value indicates better endothelial function (19). Simultaneously, EndoPAT could automatically calculate arterial stiffness, which was expressed as the augmentation index (AIx), based on radial pulse waves. Since heart rate may affect arterial stiffness, arterial stiffness was expressed by AIx75, which was corrected at a heart rate of 75 beats per minute (25).

### Biological Measurements

Fasting vein blood samples were collected in the morning following the PSG. Whole blood cell analysis, liver function, renal function, plasma glucose, uric acid, glycated hemoglobin (HbA1C), and serum lipid levels were directly measured in accredited laboratories using standard techniques within an hour. We obtained plasma and serum samples after centrifugation within half an hour at 3,000 g at 4°C for 15 min; these samples were then stored at −20°C for <1 week and transferred to −80°C until the assay was performed within half a year. Serum tumor necrosis factor-alpha (TNF-α) levels were measured using the IMMULITE 1000 Automatic Chemiluminescence Immunoassay (Siemens Medical, USA). Serum vascular cell adhesion molecule (VCAM), endothelin-1 (ET-1), and plasma vascular endothelial growth factor (VEGF) were measured using the Human Svcam-1/CD106 Quantikine ELISA Kit (RD, USA), the Endothelin-1 Quantikine ELISA kit (RD, USA), and the Human VEGF Quantikine ELISA Kit (RD, USA), respectively, according to the instruction books.

### Statistical Analysis

All data were analyzed using SPSS 22.0 and GraphPad Prism8 (GraphPad Software Inc., La Jolla, USA). The normality of the variables was tested with the Kolmogorov–Smirnov test. An unpaired, two-tailed *t*-test and a Chi-square analysis were used for comparisons between groups. The non-parametric Mann–Whitney *U*-test was used when the data were not normally distributed. The results were expressed as the mean ± standard deviations or as the median with the interquartile range. Pearson's or Spearman's methods were used to analyze the correlation between the laboratory data and PSG results. A partial correlation analysis was used to control for confounding factors and analyze the relationship between PSG and laboratory data. An ANOVA was used for multi-group comparisons, and the Kruskal–Wallis test was used for non-parametric multi-group comparisons. The confounding factors (biomarkers, vascular endothelial function, and arterial stiffness) were analyzed by a multiple linear regression. *P* < 0.05 was considered statistically significant.

## Results

**Figure 1:**
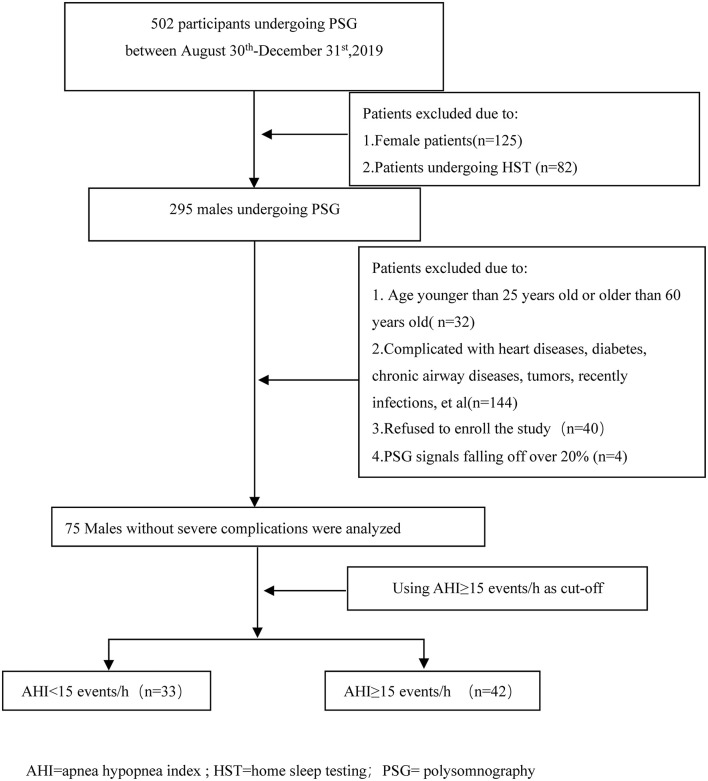
Flow diagram of the participants during the study. AHI, apnea hypopnea index; HST, home sleep testing; PSG, polysomnography.

A total of 502 patients underwent sleep respiratory monitoring in the sleep centers from August 30th, 2019 to December 31st, 2019. After excluding patients who did not meet the inclusion criteria or refused to join the research, or in whom more than 20% of polysomnography signals were lost, 75 subjects were enrolled in this study (Figure 1). The average patient age was 39.21 ± 8.34 years, and the average body mass index (BMI) was 26.68 ± 3.22 kg/m^2^. There were 26 (34.7%) hypertensive patients and 19 (25.3%) current smokers. The average sleep time was 452.87 ± 37.27 min and the average sleep efficiency was 91.95 ± 7.15%. The average AHI was 28.20 ± 26.59 events/h. The average SpO_2_ was 96.33 ± 2.11%. Using an AHI of ≥15 events/h as the cutoff, there were 33 participants with an AHI of <15 events/h and 42 participants with an AHI of ≥15 events/h.

The high-sensitivity C-reactive protein (hsCRP) levels ranged from 0.17 to 25.83 mg/L, but only two (2.7%) cases had hsCRP levels higher than the normal range. The average TNF-α level was 8.20 ± 3.63 pg/ml, and ET-1 levels ranged from 0.089 to 2.41 pg/ml. VCAM levels ranged from 346.48 to 1168.02 ng/ml, VEGF levels ranged from 3.91 to 217.91 pg/ml, the RHI ranged from 1.12 to 3.13, and the AIx75 ranged from −38 to 42%.

### The Relationship Between OSA Severity, Endothelial Function, and Arterial Stiffness

**Figure 2:**
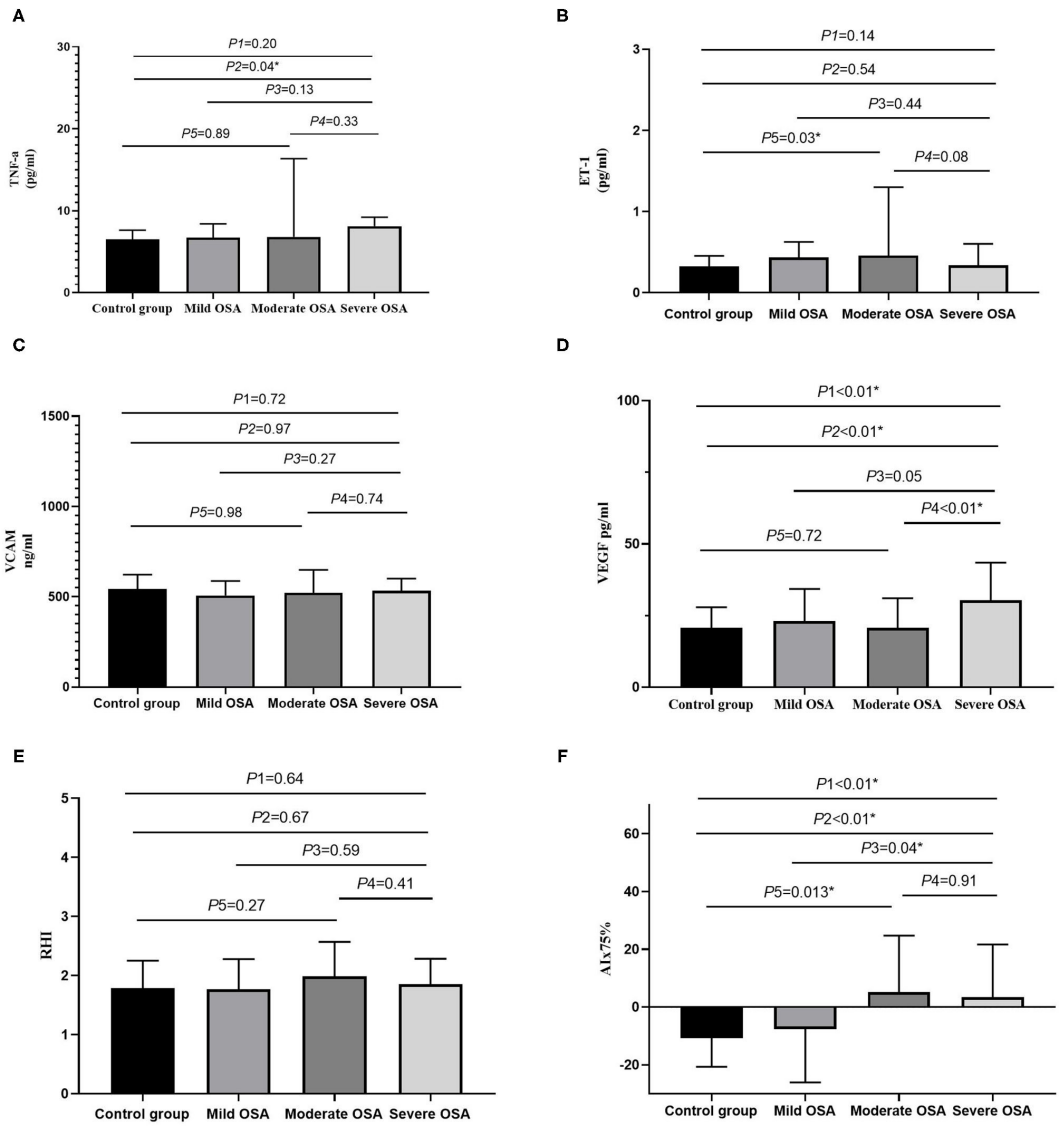
The bar diagrams showing markers in patients with OSA. **(A)** The relationship between TNF-α and OSA severity. **(B)** The relationship between ET-1 and OSA severity. **(C)** The relationship between VCAM and OSA severity. **(D)** The relationship between VEGF and OSA severity. **(E)** The relationship between RHI and OSA severity. **(F)** The relationship between AIx75 and OSA severity. AI, arousal index; AHI, apnea hypopnea index; AIx75, augmentation Index@75 beats/minute; BMI, body mass index; ET-1, endothelin-1; GLU, glucose; HbA1C, hemoglobin A1C; LSpO_2_, lowest pulse arterial oxygen saturation; RHI, reactive hyperaemia index; SpO_2_, oxygen saturation; T90, percentage of sleep time with oxygen saturation <90%; TNF-α, tumor necrosis factor alpha; VCAM, vascular cell adhesion molecule; VEGF, vascular endothelial growth factor; *P*1, four groups comparison; *P*2, severe OSA vs. control group; *P*3, severe OSA vs. mild OSA; *P*4, severe OSA vs. moderate OSA; *P*5, moderate OSA vs. control group; *P < 0.05.

Table 1 presents the demographic, clinical, and laboratory data of the participants. Patients with an AHI of ≥15 events/h had higher hsCRP, VEGF, and AIx75 values than the control group did; however, no significant differences in TNF-α, ET-1, VCAM, or RHI values were observed between the two groups (*P* > 0.05). Table 2 shows that VEGF and AIx75 values were significantly and gradually elevated whereas there was no statistical association of TNF-α, ET-1, VCAM, or RHI with OSA severity. *Post-hoc* comparisons (Figure 2) revealed that severe patients had higher TNF-α, VEGF, and AIx75 values than those of the control group, higher VEGF levels than those of the moderate patients, and higher AIx75 values than those of the mild patients. Moderate OSA patients had higher ET-1 and AIx75 values than those of the control group. No significant differences in VCAM or RHI were found between the different OSA severity groups in the *post-hoc* tests.

**Table 1:** Clinical data and laboratory data of participants.

	**All**	**AHI <15 events/h**	**AHI ≥15 events/h**	***P*-value**
*n*	75	33	42	
Age (years)	39.21 ± 8.34	38.61 ± 7.67	39.69 ± 8.90	0.58
BMI (kg/m^2^)	26.68 ± 3.22	25.60 ± 3.00	27.53 ± 3.17	<0.01
Hypertension (*n*)	26	5	21	<0.01
Current smokers (*n*)	19	5	14	0.072
Rhinitis (*n*)	26	12	14	0.71
Stage N1 (%/TST)	7.72 ± 5.16	7.49 ± 4.56	7.90 ± 5.63	0.73
Stage N2 (%/TST)	41.46 ± 11.2	38.76 ± 10.37	43.58 ± 11.50	0.06
Stage N3 (%/TST)	25.31 ± 12.45	26.98 ± 10.23	24 ± 13.92	0.31
Stage REM (%/TST)	17.20 ± 6.11	17.91 ± 6.70	16.64 ± 5.62	0.38
Stage Wake (%/TST)	8.05 ± 7.15	8.57 ± 6.67	7.64 ± 7.57	0.58
AI (events/h)	8.4 (5, 14.6)	6.3 (4.15, 9.2)	12.35 (6.48, 21.65)	<0.01
AHI (events/h)	16.6 (5.90, 51.30)	3.4 (0.8, 8.3)	48 (25.58, 64.83)	<0.01
ODI (events/h)	15.50 (1.80, 47.30)	1.5 (0.65, 4.95)	41.3 (20.95, 61.65)	<0.01
Mean SpO_2_ (%)	96.33 ± 2.11	97.3 ± 1.36	95.57 ± 2.30	<0.01
LSpO_2_ (%)	85.59 ± 7.90	91.55 ± 3.73	80.90 ± 7.10	<0.01
T90 (%)	0.1 (0, 1.10)	0 (0, 0)	0.8 (0.275, 2.33)	<0.01
HB (g/L)	155.83 ± 11.26	155.15 ± 10.93	156.36 ± 11.62	0.65
ALT (U/L)	32 (20, 54)	28 (19, 40.5)	39 (25.5, 56)	0.02
Cr (μ mol/L)	76.93 ± 9.60	79.30 ± 8.97	75.07 ± 9.78	0.058
GLU (mmol/L)	4.9 (4.7, 5.3)	4.8 (4.5, 5.1)	5.1 (4.8, 5.5)	<0.01
HBA1C (%)	5.4 (5.1, 5.6)	5.2 (5.05, 5.5)	5.5 (5.3, 5.6)	<0.01
TG (mmol/L)	1.46 (1.04, 2.39)	1.14 (0.91, 1.89)	1.72 (1.36, 3.33)	<0.01
TC (mmol/L)	4.81 ± 0.77	4.73 ± 0.78	4.87 ± 0.76	0.41
LDLC (mmol/L)	3.11 ± 0.72	3.12 ± 0.68	3.10 ± 0.76	0.88
HDL (mmol/L)	1.10 ± 0.26	1.17 ± 0.24	1.05 ± 0.26	0.03
UA (μmol/L)	405.80 ± 82.98	395.85 ± 71.94	413.62 ± 90.81	0.36
hsCRP (mg/L)	1.17 (0.69, 2.01)	0.8 (0.5, 1.33)	1.64 (0.85, 2.44)	<0.01
TNF-α (pg/ml)	7.2 (6, 8.8)	6.6 (5.8, 8)	7.9 (6.08, 9.23)	0.07
ET-1 (pg/ml)	0.36 (0.26, 0.63)	0.36 (0.21, 0.54)	0.36 (0.26, 0.68)	0.46
VCAM (ng/ml)	530.36 (461.35, 617.61)	520.51 (451.62, 619.35)	532.48 (477.84, 610.27)	0.51
VEGF (pg/ml)	25.47 (19.88, 34.26)	22.28 (17.09, 29.86)	28.27 (21.28, 35.45)	0.03
AIx75 (%)	−4 (−14, 8)	−12 (−18.5, 0)	−1 (−8, 17.25)	<0.01
RHI	1.84 ± 0.48	1.78 ± 0.48	1.89 ± 0.48	0.32

*Data presented as mean ± SD or the median with interquartile range. AI, arousal index; AHI, apnea hypopnea index; ALT, alanine transaminase; AIx75, augmentation Index@75 beats/minute; BMI, body mass index; Cr, Creatinine; ET-1, endothelin-1; GLU, glucose; HB, hemoglobin; HbA1C, hemoglobin A1C; HDL, high density lipoprotein; hsCRP, high sensitivity C reactive protein; LDLC, low density lipoprotein; ODI, oxygen desaturation index; LSpO_2_, lowest pulse arterial oxygen saturation; REM, rapid eye movement; RHI, reactive hyperaemia index; SpO_2_, oxygen saturation; T90, percentage of sleep time with oxygen saturation <90%; TC, total cholesterol; TG, triglyceride; TST, total sleep time; TNF-α, tumor necrosis factor alpha; UA, uric acid; VCAM, vascular cell adhesion molecule; VEGF, vascular endothelial growth factor*.

**Table 2:** The clinical data and laboratory data in different severity obstructive sleep apnea.

	**Normal**	**Mild OSA**	**Moderate OSA**	**Severe OSA**	***P-*value**
*N*	18	15	13	29	
Age (years old)	38.28 ± 8.58	39.00 ± 6.70	41.62 ± 10.24	38.83 ± 8.28	0.72
BMI (kg/m^2^)	24.89 ± 2.97	24.46 ± 2.89	26.46 ± 1.84	28.01 ± 3.54	0.01
Hypertension (*n*)	3	2	3	18	<0.01
Current smoker (*n*)	3	2	2	12	0.09
AHI (events/h)	0.85 (0.6, 1.8)	8.3 (6.6, 11.5)	19.8 (16.20, 24.75)	57.7 (46.4, 69.8)	<0.01
AI (events/h)	5.25 (3.3, 8.2)	7 (4.7, 9.9)	7.8 (5.5, 11.45)	16 (7.4, 27.5)	<0.01
Mean SpO_2_ (%)	97.428 ± 1.43	97.15 ± 1.31	96.10 ± 2.01	95.33 ± 2.41	<0.01
LSpO_2_ (%)	93.00 ± 3.12	89.8 (± 3.75)	84.54 ± 5.83	79.28 ± 7.11	<0.01
T90 (%)	0 (0, 0)	0 (0, 0.10)	0.30 (0.05, 0.80)	1.4 (0.35, 3.4)	<0.01
TNF-α (pg/ml)	6.51 (5.85, 7.63)	6.7 (5.5, 8.4)	6.8 (5.65, 16.35)	8.1 (6.4, 9.2)	0.20
VEGF (pg/ml)	20.68 (18.88, 27.87)	23.08 (13.49, 34.26)	20.68 (17.89, 31.06)	30.263 (26.27, 43.44)	<0.01
ET-1 (pg/ml)	0.33 (0.20, 0.46)	0.43 (0.22, 0.63)	0.46 (0.31, 1.3)	0.34 (0.26, 0.60)	0.14
VCAM (ng/ml)	543.42 (472.05, 622.52)	507.30 (437.69, 587.33)	522.15 (450.93, 649.52)	533.30 (486.65, 600.91)	0.72
AIx75 (%)	−15 (−18.25, −1.75)	−11 (−22, 2)	−1 (−8.5, 20)	−1 (−8, 17.5)	<0.01
RHI	1.78 ± 0.46	1.77 ± 0.51	1.98 ± 0.58	1.85 ± 0.44	0.64

*Data presented as mean ± SD or the median with interquartile range. AI, arousal index; AHI, apnea hypopnea index; AIx75, augmentation Index@75 beats/minute; BMI, body mass index; ET-1, endothelin-1; GLU, glucose; HbA1C, hemoglobin A1C; LSpO_2_, lowest pulse arterial oxygen saturation; RHI, reactive hyperaemia index; SpO_2_, oxygen saturation; T90, percentage of sleep time with oxygen saturation <90%; TNF-α, tumor necrosis factor alpha; VCAM, vascular cell adhesion molecule; VEGF, vascular endothelial growth factor*.

### Relative Associations Between OSA, Endothelial Function, and Arterial Stiffness

We further explored the relative associations between endothelial function, arterial stiffness, and OSA using a bivariate analysis. TNF-α was positively associated with BMI (*r* = 0.356, *P* < 0.05), ODI (*r* = 0.277, *P* < 0.05), and T90 (*r* = 0.306, *P* < 0.05) and negatively correlated with the mean SpO_2_ (*r* = −0.389, *P* < 0.05) and LSpO_2_ (*r* = −0.320, *P* < 0.05). ET-1 was negatively correlated with the percentage in stage N1 (*r* = −0.251, *P* < 0.05). VEGF was positively correlated with BMI (*r* = 0.250, *P* < 0.05), the percentage in stage N3 (*r* = 0.237, *P* < 0.05), AHI (*r* = 0.349, *P* < 0.05), ODI (*r* = 0.348, *P* < 0.05), and T90 (*r* = 0.265, *P* < 0.05) and was negatively correlated with the percentage in the wake stage (*r* = −0.254, *P* < 0.05), the mean SpO_2_ (*r* = −0.259, *P* < 0.05), and LSpO_2_ (*r* = −0.268, *P* < 0.05). RHI was only positively correlated with the percentage in the REM stage (*r* = 0.31, *P* = 0.007). AIx75 was positively correlated with age (*r* = 0.559, *P* < 0.0001), AHI (*r* = 0.367, *P* = 0.001), ODI (*r* = 0.298, *P* = 0.009), AI (*r* = 0.307, *P* = 0.007), and T90 (*r* = 0.288, *P* = 0.012) and was negatively correlated with LSpO_2_ (*r* = 0.305, *P* = 0.008).

**Figure 3:**
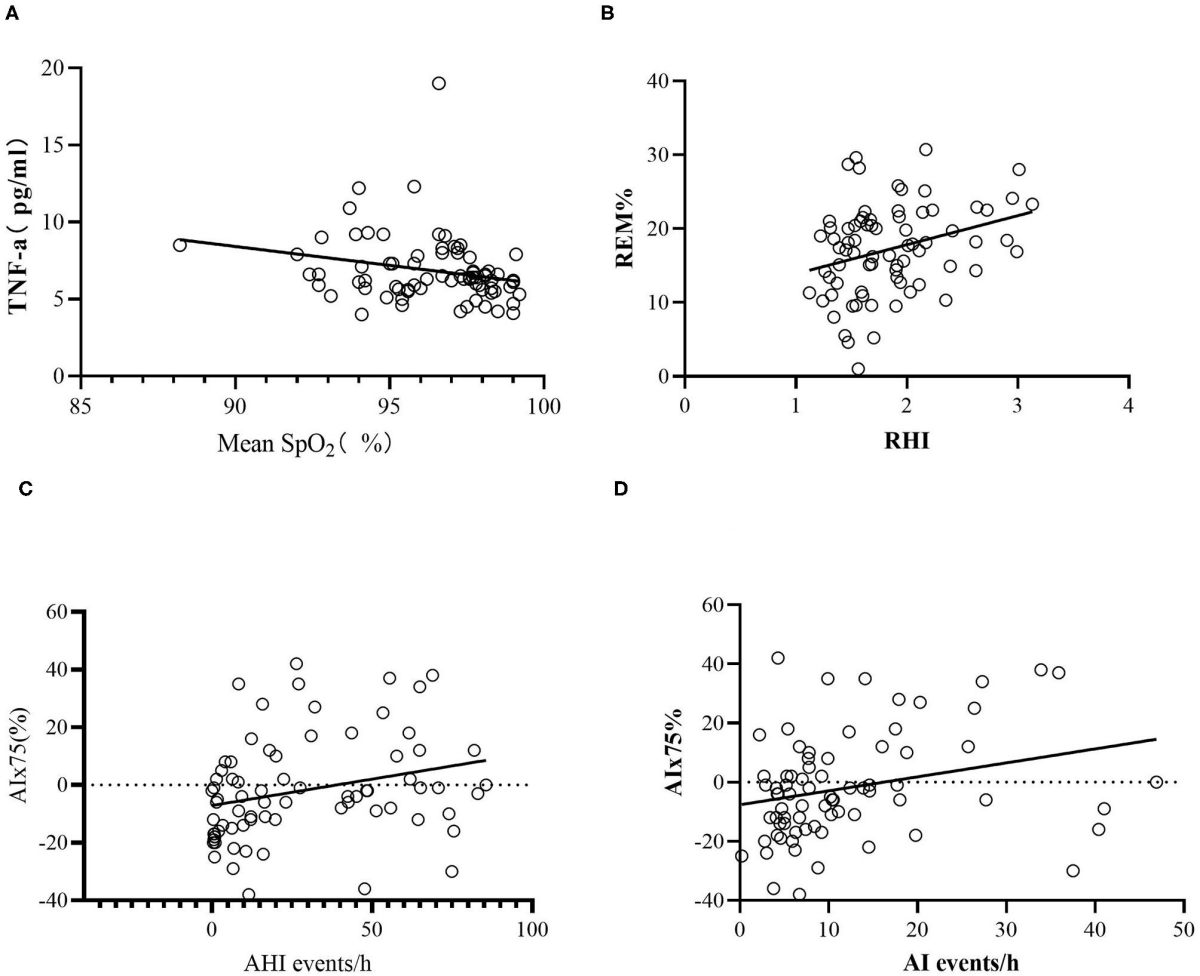
Scatter plots of laboratory data. **(A)** The relative associations between TNF-α and mean SpO_2_. **(B)** The relative associations between REM% and RHI. **(C)** The relative associations between AIx75 and AHI. **(D)** The relative associations between AIx75 and AI. AHI, apnea hypopnea index; AI, arousal index; AIx75, augmentation Index@75 beats/minute; SpO_2_, oxygen saturation; TNF-α, tumor necrosis factor alpha; REM%, the percentage of stage rapid eye movement in total sleep time; RHI, reactive hyperaemia index.

After controlling for age, BMI, rhinitis, hypertension, smoking, glucose level, HbA1C, hemoglobin (Hb), creatinine (Cr), total cholesterol (TC), triglycerides (TG), low-density lipoprotein (LDLC), and high-density lipoprotein (HDL), we found that TNF-α was negatively associated with the mean SpO_2_ (*r* = −0.258, *P* = 0.043; Figure 3). No significant correlations between hsCRP, VEGF, ET-1, VCAM, and the parameters of PSG were observed. RHI was also positively correlated with the percentage in the REM stage (*r* = 0.306, *P* = 0.016) but was not correlated with other parameters of PSG or clinical characteristics of OSA. AIx75 remained positively correlated with the AI (*r* = 0.289, *P* = 0.023), but the correlation between AIx75 and AHI was not statistically significant (*r* = 0.248, *P* = 0.052; Figure 3).

We used a multiple step regression to analyze the relationships between TNF-α, RHI, and AIx75 and age, BMI, AHI, AI, PSG parameters (mean SpO_2_, LSpO_2_, T90, percentages in stages N1, N2, N3, REM, and wake), and cardiovascular risk factors (smoking, hypertension, blood glucose, HbA1C, TC, TG, HDL, and LDLC). The results showed that TNF-α was positively correlated with the percentage in stage N1 (*B* = 0.166, *P* = 0.025) and was negatively correlated with the mean SpO_2_, HDL, and TG (*B* = −0.398, *P* = 0.034; *B* = −5.263, *P* = 0.002; *B* = −0.657, *P* = 0.013, respectively). TNF-α was not significantly correlated with age, BMI, hypertension, smoking, AHI, blood glucose, or HbA1c. RHI was positively correlated with hypertension and the percentage in the REM stage (*B* = 0.275, *P* = 0.013; *B* = 0.026, *P* = 0.003, respectively) but was not significantly correlated with age, BMI, smoking, AHI, SpO_2_, LSpO_2_, T90, blood glucose, or HbA1c. AIx75 was positively correlated with age, AI, and TC (*B* = 1.201, *P* < 0.001; *B* = 0.547, *P* = 0.001; *B* = 6.036, *P* = 0.004, respectively) but was not significantly correlated with BMI, smoking, AHI, SpO_2_, LSpO_2_, T90, blood glucose, HbA1C, TG, LDLC, or HDL.

## Discussion

Our study comprehensively assessed systemic inflammatory biomarkers and vascular endothelial function in Chinese middle-aged men with moderate and severe OSA without severe complications. We found that these patients had only low levels of systemic inflammatory biomarkers and vascular endothelial biomarkers, which were mainly manifested by elevated hsCRP, TNF-α, and VEGF levels. After controlling for the confounding factors, TNF-α was negatively correlated with the average SpO_2_ but was not correlated with AHI. RHI was still positively correlated with the REM ratio but was not with the average SpO_2_, LSpO_2_, or AHI values. There was a correlation between AIx75 and AI, but the correlation between AIx75 and AHI was not statistically significant. The multiple stepwise regression analysis showed that TNF-α was positively correlated with the percentage in stage N1 and was negatively correlated with the average SpO_2_, HDL, and TG values. RHI was positively correlated with hypertension and the percentage in the REM stage whereas AIx75 was positively correlated with age, AI, and TC.

Although many studies on systemic inflammatory biomarkers and the vascular endothelial function of OSA have been reported, most such studies did not exclude patients with related cardiovascular diseases and diabetes or only assessed a single marker, which fails to effectively compare the value of various markers to evaluate OSA patients. Although we did not find a direct association between the AHI and RHI detected by PAT testing, we found that some clinical significance could be determined from measuring hsCRP, TNF-α, VEGF, arterial stiffness, and vascular endothelial function in patients with moderate and severe OSA. Additionally, the results noted that inflammatory indicators are not only associated with hypoxia induced by OSA but also with sleep structural disorders, which confirmed that hypoxia and sleep fragmentation were two mechanisms by which OSA leads to abnormal vascular endothelial function and changes in arterial stiffness.

Currently, there are limited studies measuring endothelial function using the PAT technique in OSA patients. However, our research did not identify an association between RHI and OSA severity. In fact, the role of RHI originating from PAT in OSA patients remains uncertain. Our results were similar with those of some previous studies. Most of these studies were performed in OSA patients with complications including obesity, diabetes, and ischemic stroke (18, 26, 27). However, other studies had distinct results. For example, they noted that RHI was not only associated with OSA severity but also with the level of hypoxia and sleep fragmentation (7, 8, 17, 28). Farooqui et al. compared RHI, FMD, and CIMT in 20 cases with OSA and 20 control cases without any complications and found that patients with moderate and severe OSA had lower FMD and RHI and a greater CIMT, but these variables were not correlated with OSA severity (8). Zhang et al. found that the RHI of children with OSA was significantly lower than that of simple snore patients and was independently correlated with age, BMI, and the respiratory AI using a stepwise regression (28). A systematic review of 12 studies that applied PAT and sleep breathing monitoring in 730 cases without cardiocerebrovascular diseases showed that endothelial function was related to severe OSA. In the subgroup analysis of patients with hypertension, obesity, and diabetes, the correlations between endothelial function and severe OSA were not significant (7).

Additionally, the roles of PAT and FMD in evaluating the endothelial function of OSA were inconsistent. Previous studies were mainly performed using FMD. FMD was used to assess the changes in the dilation of the diameter resulting from reactive hyperemia using B type ultrasound whereas PAT was performed to detect the volume variation of the digital vascular bed using the PAT technique. The principle of both methods involves measuring the change of blood vessel volume caused by nitric oxide (NO) released by the blood vessels after pressure (29). However, FMD measures vessels in the brachial artery and PAT measures the blood vessels at the end of the finger, which represent the lesions of the capillaries. Also, recent studies found that lower-limb FMC responses were blunted when endothelial-derived hyperpolarizing factors or prostaglandin production was inhibited which may explain the different results of FMD and PAT (30). FMD repeatability is relatively low and requires skilled operators to perform. Conversely, PAT is performed automatically by a machine and thus has high repeatability and little correlation with the operator. The Framingham Heart Study also found no correlation between PAT-RHI and FMD, suggesting that vascular endothelial injury in different parts may provide different information for predicting cardiovascular disease, but injuries in all parts can predict cardiovascular disease (31, 32). Studies have shown that when the RHI is 1.35, the sensitivity and specificity of detecting endothelial dysfunction in the coronary arteries are 80% and 85%, respectively (13). PAT has been shown to predict cardiovascular disease in very few prospective studies. PAT was tested in a group of 270 patients and was found to be an independent predictor of cardiovascular events after 7 years of follow-up (11). The RHI was also found to be an independent predictor of cardiovascular events and to improve the predictive value of the Framingham risk score in a 2.8-year follow-up study of 528 patients with a high risk of cardiovascular disease (33). Currently, few relevant studies have reported on the ability of PAT to predict cardiovascular disease in patients with OSA.

Our research revealed that RHI was correlated with the ratio of time in the REM stage even after controlling for confounding factors including age, BMI, smoking, and metabolic factors. The association between PAT and the ratio of time in the REM stage has not been reported until now. Previous studies have reported that REM sleep was linked to vascular endothelial dysfunction. The study by Cooper et al. in 100 non-shift adults, of whom 52% had OSA, found that a decrease in FMD was associated with an increase in the Pittsburgh sleep quality index, prolonged REM sleep latent time, and a reduced REM sleep ratio (34). Furthermore, a multicenter population-based cross-sectional study also found that REM sleep deprivation was associated with greater risk of cardiovascular mortality in middle-aged and older adults (35). However, the reason for the association between vascular endothelial dysfunction and REM deprivation was unclear. REM sleep has been demonstrated to play an important role in studying, memory, and daytime function (36). The NO in the brain contributed to REM sleep (37) while FMD and PAT measure vascular function through NO-mediated vascular dilation. An animal study showed that REM sleep deprivation could induce endothelial dysfunction and hypertension through the endothelial NO synthase pathway (38). Some animal studies also showed that REM sleep deprivation could create oxidative stress and reactive oxygen species which may contribute to endothelial dysfunction (39, 40).

Our research also indicates that RHI was positively related to hypertension. Hypertension is a risk factor for many cardiovascular diseases. In theory, hypertensive patients would have a lower RHI. The study of Framingham cohorts with 1,957 subjects found that RHI was negatively correlated with hypertension in age- and sex-adjusted models but not related with hypertension in stepwise multivariable linear regression models (12). A study of a Brazilian cohort in 2014 showed that systolic pressure was positively associated with RHI and that patients using hypotensive drugs had a lower RHI (10). Another study in chronic kidney disease patients reported a positive association between systolic pressure and RHI, which was similar with our results (41). Consequently, the association between RHI and hypertension is uncertain. We speculated that the repeatability of RHI was poor in hypertensive patients because sympathetic activity changes induced increased heart rate variation (42).

Many studies have suggested that arterial stiffness is an early marker of atherosclerosis. At present, most studies on the relationship between OSA and arterial stiffness are mainly based on pulse wave velocity analysis. Founded on the theory of arterial reflected waves, increasing attention has been paid to the reflected wave enhancement index based on pulse wave shape analysis. Increased AIx has been shown to predict future cardiovascular events (43), and the radial AIx can be used as an alternative to the marker of primary prevention of atherosclerosis (44). A meta-analysis showed that the vascular AIx of moderate and severe OSA increased significantly compared with that of the control group (16), and CPAP can improve the AIx of OSA (21).

Our study found that after controlling for confounding factors, AIx75 was still associated with the AI of OSA sleep fragmentation, suggesting that OSA sleep fragmentation is one of the reasons for atherosclerosis independent of age, BMI, hypertension, and other factors. The partial correlation between AIx75 and AHI did not reach statistical significance, which may be related to the small sample size. At present, there are insufficient studies on the application of the peripheral vascular tension technique to measure arterial stiffness in OSA patients. A study assessed arterial stiffness in 37 patients with ischemic stroke using PAT technology and found that even after controlling for sex, BMI, hypertension, and diabetes, AIx was correlated with the AI and mean oxygen saturation (26). Theorell-Haglow et al. measured radial artery stiffness in OSA patients using the flat tension method and found that OSA severity was related to the radial artery stiffness AIx75 (45). The results of this study are consistent with those above-mentioned previous studies. The PAT technique is simple to conduct, highly repeatable, and can be used routinely to assess the arterial stiffness of OSA patients, which is beneficial for detecting early changes of atherosclerosis in OSA patients.

Our study has some limitations. Firstly, we only performed this study in males, so the results cannot be generalized to all patients with OSA. However, males comprise the majority of OSA patients, so these results can still help a large proportion of patients. Secondly, the PAT value is influenced by many factors including the effects of environment, stress, diet, age, BMI, exercise, smoking, and disease. To detect the evaluation role of PAT in OSA patients, we only included OSA males without severe complications. All of the patients were advised to avoid rigorous exercises within 24 h, smoking, tea, and coffee. PAT values were measured at least half an hour before or after blood drawing. However, changes in the sleep environment and stress due to blood puncture may inevitably have affected the results of the vascular endothelial function. Additionally, the small sample size of this study may have affected the results. In the future, it is necessary to further expand the sample size and observe the changes of treatment response to verify our results.

## Conclusion

In conclusion, our assessments including systemic inflammatory indicators, endothelial function, and arterial stiffness showed that moderate and severe OSA patients without severe complications only had low levels of inflammation and vascular endothelial injury markers, which were mainly manifested by hsCRP, TNF-α, and VEGF, among which TNF-α was independently associated with OSA. AIx75, the arterial stiffness marker measured by PAT, is closely related to OSA. The relationship between RHI and OSA requires further investigation. Endothelial function and arterial stiffness should be measured to comprehensively assess the early vascular damages by OSA without severe complications in clinical practice to provide more evidence for the primary prevention of coronary heart disease and treatment response assessment.

## Data Availability Statement

The original contributions presented in the study are included in the article/supplementary material, further inquiries can be directed to the corresponding author/s.

## Ethics Statement

The studies involving human participants were reviewed and approved by the Ethics Commission of Peking Union Medical College Hospital, Chinese Academy of Medical Sciences, China (protocol number JS-2013). The patients/participants provided their written informed consent to participate in this study.

## Author Contributions

JL designed the study, collected and analyzed the data, and drafted the manuscript. XW collected and analyzed the data. YX supervised the study and reviewed and edited the manuscript. ZG and LZ performed the biological measurements experiments. WC, LS, JG, and RH collected the clinical and polysomnography data. All authors contributed to the article and approved the submitted version.

## Funding

This work was supported by National Natural Science Foundation of China (81570085) and the National Key Research and Development Projects of China (No. 2018YFC1315103).

## Conflict of Interest

The authors declare that the research was conducted in the absence of any commercial or financial relationships that could be construed as a potential conflict of interest.

## Publisher's Note

All claims expressed in this article are solely those of the authors and do not necessarily represent those of their affiliated organizations, or those of the publisher, the editors and the reviewers. Any product that may be evaluated in this article, or claim that may be made by its manufacturer, is not guaranteed or endorsed by the publisher.
